# EEG-Based Quantification of Cortical Current Density and Dynamic Causal Connectivity Generalized across Subjects Performing BCI-Monitored Cognitive Tasks

**DOI:** 10.3389/fnins.2017.00180

**Published:** 2017-05-17

**Authors:** Hristos Courellis, Tim Mullen, Howard Poizner, Gert Cauwenberghs, John R. Iversen

**Affiliations:** ^1^Swartz Center for Computational Neuroscience, University of California, San DiegoSan Diego, CA, United States; ^2^Department of Bioengineering, University of California, San DiegoSan Diego, CA, United States; ^3^Institute for Neural Computation, University of California, San DiegoSan Diego, CA, United States

**Keywords:** brain-computer interface (BCI), electroencephalography (EEG), causality analysis, source localization, LORETA, independent component analysis, motor activity, spatial reach and saccade

## Abstract

Quantification of dynamic causal interactions among brain regions constitutes an important component of conducting research and developing applications in experimental and translational neuroscience. Furthermore, cortical networks with dynamic causal connectivity in brain-computer interface (BCI) applications offer a more comprehensive view of brain states implicated in behavior than do individual brain regions. However, models of cortical network dynamics are difficult to generalize across subjects because current electroencephalography (EEG) signal analysis techniques are limited in their ability to reliably localize sources across subjects. We propose an algorithmic and computational framework for identifying cortical networks across subjects in which dynamic causal connectivity is modeled among user-selected cortical regions of interest (ROIs). We demonstrate the strength of the proposed framework using a “reach/saccade to spatial target” cognitive task performed by 10 right-handed individuals. Modeling of causal cortical interactions was accomplished through measurement of cortical activity using (EEG), application of independent component clustering to identify cortical ROIs as network nodes, estimation of cortical current density using cortically constrained low resolution electromagnetic brain tomography (cLORETA), multivariate autoregressive (MVAR) modeling of representative cortical activity signals from each ROI, and quantification of the dynamic causal interaction among the identified ROIs using the Short-time direct Directed Transfer function (SdDTF). The resulting cortical network and the computed causal dynamics among its nodes exhibited physiologically plausible behavior, consistent with past results reported in the literature. This physiological plausibility of the results strengthens the framework's applicability in reliably capturing complex brain functionality, which is required by applications, such as diagnostics and BCI.

## Introduction

Dynamic intra-cortical interactions offer a wealth of information that can be used to develop an understanding of brain functionality and quantify the effects of brain pathologies on brain functionality. Traditional static functional characteristics of individual brain regions, and dynamic interactions within a network of brain regions (Moeller et al., 2015) offer limited insight into brain function as they typically do not examine the causality among these interactions. The advent of Granger-causal analysis provided a framework to quantify the asymmetric causal interactions between regions of the cortex through specific frequency bands, which hold functional significance for those regions (Barnett and Seth, 2014; Seth et al., 2015), and has shed light on a large number of network-related disorders, such as Alzheimer's and Parkinson's Disease (Pievani et al., 2014). However, this connectivity information is not limited in use to diagnostics, as an increasing number of brain-computer interface (BCI) applications are utilizing connectivity features for intent discrimination when conducting BCI-related tasks (Zhang et al., 2012; Kabbara et al., 2016).

While a potential wealth of information is available in intra-cortex interactions, a systematic algorithmic approach that allows for reliable generalization of cortical network dynamics across test subjects in a subject group is not readily available. Many sophisticated tools exist (Linder et al., 2011; Niso et al., 2013; Barnett and Seth, 2014), which allow for both parametric and non-parametric estimation of dynamic causal interactions among neural activity signals within an individual. However, these tools exhibit various limitations, such as strict channel-space applicability or lack of accounting of common sources, thus, giving rise to spurious causal interactions within the chosen cortical network (Trongnetrpunya et al., 2015). Additional limitations exist in the source-localization algorithms applied to reconstruct cortical-source activity from the measured channel space data. Many popular algorithms stemming from Independent Component Analysis (ICA) are not able to achieve reproducible source localization outcomes across test subjects (Bell and Sejnowski, 1995). The inability to control where these algorithms localize the sources for decomposition on separate subjects presents a large problem since the sources represent nodes in a cortical network, and an ICA-based algorithm could localize a different network for every subject in a group, making group-level network comparisons difficult. Independent Component (IC) ordering is a highly non-trivial task (Hyvärinen and Oja, 2000) for which very few automated approaches exist barring manual selection, a prohibitively tedious and time consuming task for increasingly large datasets.

In this paper, we develop an algorithmic and computational framework that addresses the aforementioned issues through a multi-step, open-source pipeline of analytic tools (Delorme et al., 2011; Iversen et al., 2014). The pipeline combines the deconvolution power of ICA; the systematic modeling of the geometry of gray matter, thereby creating a common source space across subjects with the use of individually warped boundary element models (BEMs); the selectivity afforded by Cortical Low Resolution Electromagnetic Tomography (cLORETA)—a source-space based, distributed source localization algorithm; and the robustness of the Short-time direct Directed Transfer Function (SdDTF)—a causality measure, computed from Multivariate Autoregressive (MVAR) model coefficients, that addresses the spurious connectivity affecting Granger Causal methods (Korzeniewska et al., 2003).

A similar approach to source localization and connectivity analysis was proposed by Mullen et al. (2015) as a means of demonstrating that the connectivity features considered in this framework can be applied with great success in real-time brain state identification. In order to demonstrate the power afforded by this framework in group-level analysis, a BCI-related task originally introduced by Park et al. (2014) was employed, requiring subjects to reach and saccade to presented visual targets while their cortical activity was recorded using electroencephalography (EEG) as the neuroimaging modality. The approach utilized for BCI-related task is a passive-BCI approach, also described as an implicit human-computer interaction, wherein passive EEG equipment that senses the user is used to evaluate cognitive states of the user (Schmidt, 2000; George and Lecuyer, 2010). While, in this case, the closed-loop feedback elements of the task simply involve auditory and visual confirmation of reach/saccade task completion, the applicability of this approach to more active real-time closed-loop feedback BCI systems is also discussed. Although the motor activity present in the human cortex during simple planned motor actions has been carefully documented with respect to the activity of individual cortical regions (Johnson et al., 1996; Filimon, 2010; Heider et al., 2010; Glover et al., 2012), the dynamic interactions among these cortical regions during planning and execution of the task have not been documented as extensively. Park et al. were able to differentiate between spatial movement of the arm and eye by employing channel-space empirical mode decomposition, but did not characterize connectivity between cortical regions underlying these processes. The algorithmic approach implemented in this paper was first described by Iversen et al. (2014), where the method was applied to a smaller subject group. In this paper, we introduce modifications to the original pipeline in Iversen et al. (2014) including a new maximum-power heuristic that captured previously undetected connectivity involving the Supplementary Motor Area and Precuneus, regions expected to exhibit connectivity given the motor-related BCI task, and non-parametric statistical thresholding of the computed causal connectivity values. Through application of our new pipeline, described fully in the Methods section that follows, we demonstrate in the Results section the strength and specificity of the inference that can be made regarding the dynamic causal interactions present among regions of the cortex during reach/saccade planning and execution. The Discussion section illustrates the physiological plausibility of the findings reported in the Results section with reference to previous findings reported in the literature. The Conclusion section summarizes the features, the scalability, extensibility, and the applicability of the proposed algorithmic and computational framework.

## Methods

### Overview

**Figure 1:**
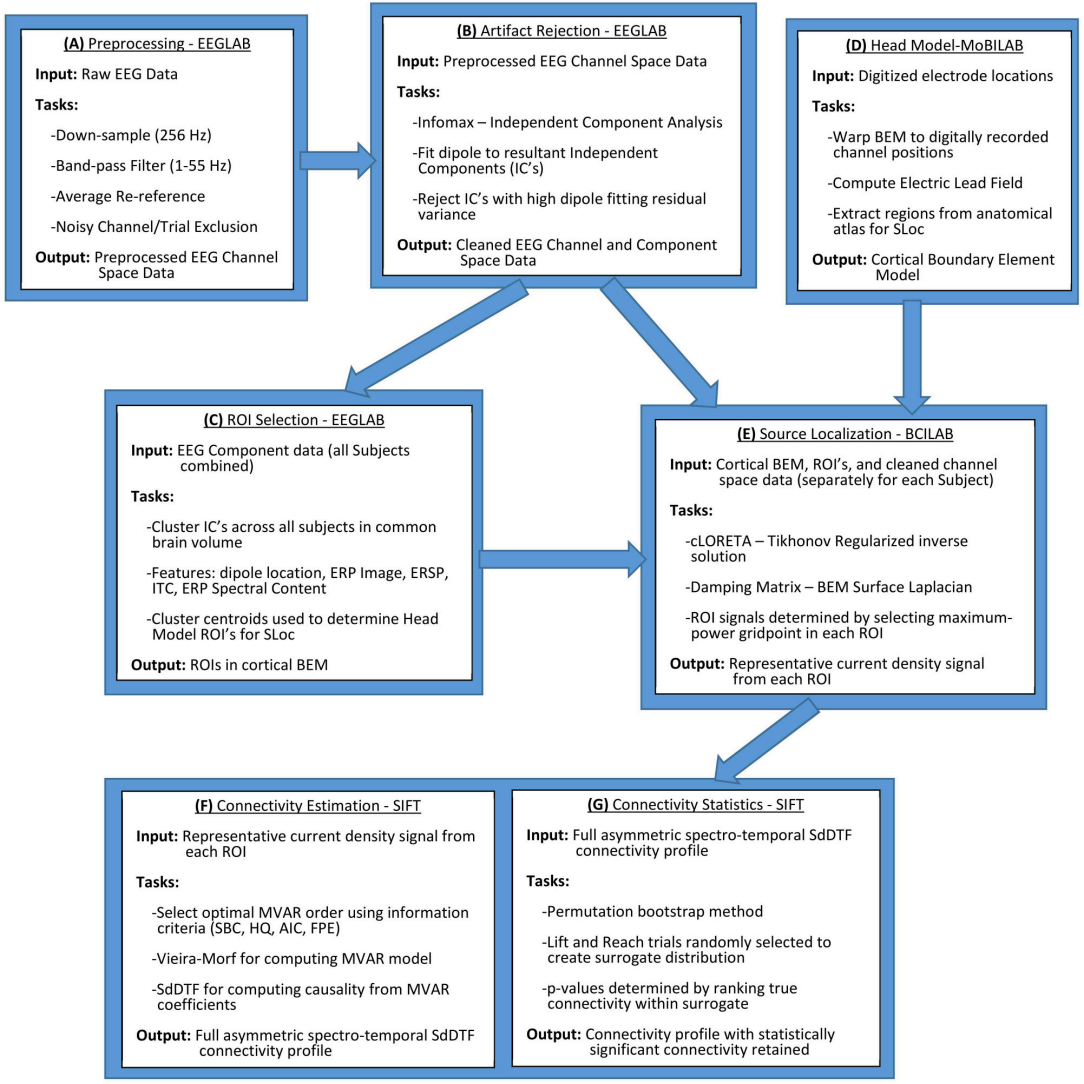
**General outline for cross-subject quantification of cortical connectivity**. Initial data processing and artifact rejection is conducted in EEGLAB (Delorme and Makeig, 2004) **(A,B)**. ROIs are selected through IC clustering, also conducted in EEGLAB **(C)**. Head model construction and Electric Lead Field calculation are conducted in MoBILAB (Ojeda et al., 2014) **(D)**. Source localization is conducted in BCILAB (Delorme et al., 2011) through an implementation of cLORETA that iteratively estimates regularization hyperparameters **(E)**. Connectivity estimation is conducted using SIFT, an extension of EEGLAB (Delorme et al., 2011) **(F,G)**. All of the above software and their internal dependencies are run in MATLAB.

Electrophysiological data were collected from human participants conducting a BCI-monitored task, and processed according to the methodology and computational tools outlined in Figure 1, using MATLAB as the computational platform. The workflow in Figure 1 was employed to extract cortical source information from a cortical network defined for all of the participants, and, subsequently, identify statistically significant spectro-temporal interactions among the nodes (sources) of this network.

### Participants and task

Ten healthy, right-handed individuals (age: 20.8 ± 2.6 years) participated in this study. The study was approved by the Human Subjects Institutional Review Board of the University of California, San Diego. Written informed consent was obtained from all subjects prior to the start of the experiment.

**Figure 2:**
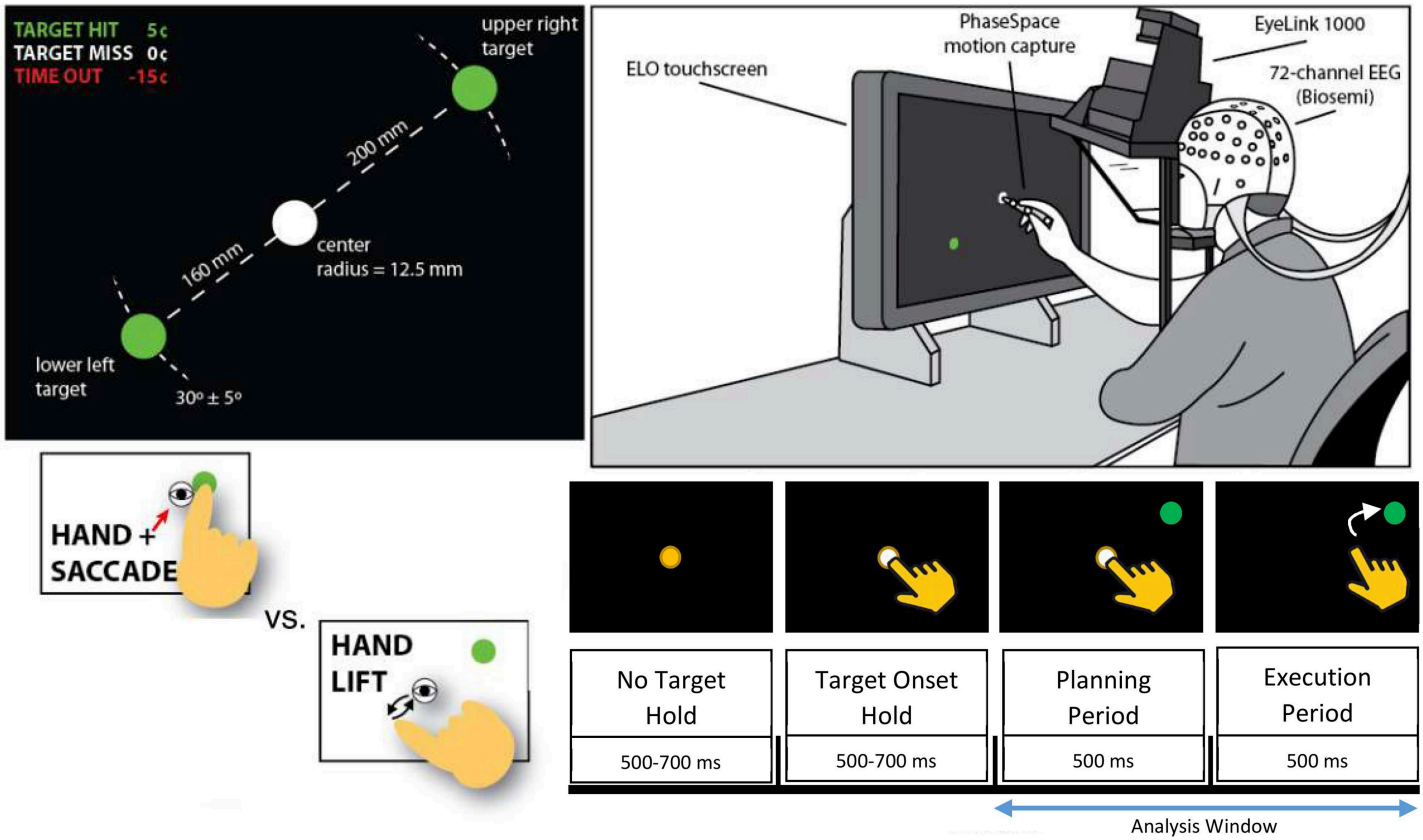
**Depiction of experimental setup from Hyvärinen and Oja (2000)**. Participants (Top Right) were placed in front of a touchscreen while brain activity was monitored by 64-channel EEG. Participants held stylus over a white fixation point in the center of the screen (Top Left) and were prompted for each trial to either lift and retouch the stylus (Control Condition) or to reach and saccade (Test Condition) to a randomly generated green target (Bottom Left). Epoch structure (Bottom Right) was identical for control and test conditions, with planning conducted while the participants remained on the fixation point with the green target present, and execution beginning when the fixation point was removed. Analysis window includes both planning and execution periods of reach/saccade or lift. Direction of the reach (upper right vs. lower left) was not discriminated since our focus was on the general causal dynamics involved in reaching rather than the encoding of spatial directionality.

The task involved a time-constrained eye movement and reach using the subject's dominant hand (right) from a target in the center of a touch-screen to a laterally-offset target as depicted in Figure 2 (Park et al., 2014). Each trial was either a control condition (lift), where the participant moved the stylus off of the central fixation dock after a brief target onset period (500–700 ms), or a test condition (reach/saccade), where the participant shifted their visual focus on and reached with the stylus to a new target that appeared in the upper right or lower left of the screen. Trials were defined as a combination of the planning period, 0 to 500 ms after the end of the target onset window, during which the subject planned either the reach/saccade or lift, and subsequent execution from 500 to 1000 ms after the target onset window, during which the subject moved their hand and reached for the new target. Each subject completed 256 trials, 96 of which were control condition trials, with the remaining 192 being test condition trials. The position of the new target randomly varied by a small angular amount (± 5 degrees) about a 30-degree direction centered at the position of the initial target—the center of the screen. Detailed description of the cognitive task performed by the participants can be found in Park et al. (2014).

### EEG recording

Scalp electroencephalographic (EEG) activity was recorded with a sampling rate of 512 Hz from 64 electrodes positioned on the scalps of the participants in an extended International 10–20 system configuration. The electrode array was grounded/referenced on a standard Driven Right Leg/Common Mode Sense (DRL/CMS) reference (Biosemi Inc., Amsterdam, The Netherlands), and each electrode was individually impedance-tested to ensure low impedance across the entire electrode array (below 5 kΩ for each electrode). The 3D position of each electrode was digitized in order to construct individualized head models for each participant (see Park et al., 2014 for details on the digitization system).

### Preprocessing and artifact rejection

Scalp electroencephalographic (EEG) channel space data preprocessing was conducted using EEGLAB (Delorme and Makeig, 2004) through a procedure outlined in Figure 1A. EEG data from each of the 10 participants were band-pass filtered, retaining frequencies from 1 to 55 Hz, and re-referenced to the channel average since the data was recorded reference-free. The data were epoched, extracting the time period of interest, which encompassed both the planning phase and the execution phase of every trial. No distinction was made between reach directions since the emphasis for this analysis was placed on the general process of reach/saccade planning and execution rather than directional discrimination and, as such, both upward and downward angled reach trials were pooled and analyzed together. The control trials (Lift) and test trials (Reach/Saccade) were analyzed separately, using a cortical network defined over the same ROIs. Differences between the control and test conditions were examined by considering differences in network dynamics between the control and test conditions during the planning and execution phases. Noise and artifact laden trials and channels were identified and removed in a semi-automated manner. The kurtosis of each channel or trial was used as the identification metric, with channels or trials exhibiting a kurtosis Z-score > +5 or < −5 being flagged and subsequently visually inspected before removal.

The epoched channel space data were decomposed into sets of maximally independent components, where independence was achieved by minimizing mutual information between components using Infomax ICA (Bell and Sejnowski, 1995) (process outlined in Figure 1B). These components represented a combination of putative effective cortical sources, muscular artifacts, ocular artifacts, and electrical activity from the heart, among other noise sources. Through a semi-automated procedure, cortical sources were defined by employing independent components that both visually, by identifying power spectra and topographical maps with dipolar cortical source character, and computationally, through dipole-fitted residual variance, corresponded to dipoles (each fitted to a dipole with low residual variance). This procedure is particularly well suited for EEG artifact removal since the electrophysiological activity of interest present in the cortex manifests detectably in the form of an electrical dipole. The linear mixing of the electric fields generated by these dipoles, and corresponding unmixing through ICA allows for very precise retention of cortical data and rejection of non-cortical artifacts. Using the weight matrix computed during ICA, the retained cortical independent components were re-projected to channel space, creating cleaned channel space electrical signals that were further downsampled to 128 Hz for use in subsequent processing.

### ROI selection methods-clustering

Cortical regions of interest (ROIs) for source localization were determined by spatially and functionally clustering the ICs aggregated across all datasets/participants into a common cortical volume, as outlined in Figure 1C. The clustering feature vector for each IC was constructed by combining information from: the topographical scalp map of the IC, the event related potential (ERP) associated with the IC and its power spectrum, the spectral power content of a rolling window Fourier Transform (FT) across the IC time-series data before ERP-type averaging, known as the Event-Related Spectral Perturbation (ERSP) of the IC, the associated phases from this rolling FT window that give the Inter-Trial Coherence (ITC) of the IC, the ERP-image constructed from concatenation of all trials for the epoched event, and the three dimensional location of the equivalent current dipole fitted to the IC (Makeig, 1993). These features were selected to ensure that the IC clusters were both spatially tight and functionally homogeneous, thus promoting the creation of clusters from the same cortical region that demonstrated similar spectro-temporal dynamics during the conducted task. PCA was used to reduce both the dimensionality of each of the above features, as well as the final feature vector for each IC to 10 dimensions corresponding to the 10 highest-eigenvalues, and, subsequent, K-means clustering of the ICs was conducted with *K* = 14 groups, whose clustering outcome yielded more spatially tight and functionally homogeneous clusters than the outcomes for *K* = 9 and *K* = 16 groups. The centroid of each cluster was computed and anatomically labeled using the Talairach atlas (Lancaster et al., 2000).

### Head model construction

The (BEMs) that defined the geometry of the cortical source space for current localization were constructed using MoBILAB (Ojeda et al., 2014), as outlined in Figure 1D. The BEM approximation for the cortex of each participant included a standardized head model—the MRI MNI Colin27 three-layer BEM comprised of scalp, skull, and cortical layers. The cortical layer, where the source localization n occurs, was discretized into a triangular mesh containing 4825 vertices, which were automatically aggregated into anatomical regions based on the MNI standard gray matter atlas, used for ROI selection. A customized head model was constructed for each of the 10 participants based on the digitized electrode positions on EEG cap conforming to the participant's scalp during the experiment. Using standard electrical conductance values for the three BEM layers (0.33 S/m Scalp, 0.022 S/m Skull, 0.33 S/m Cortex Oostendorp et al., 2000), an electrical lead field matrix was derived, relating the conduction of electric fields generated by current dipoles from the cortical mesh (bottom layer) to the top of the skin (outer layer) where the field potential is recorded by scalp electrodes. This lead field matrix, along with the discrete surface Laplacian of the cortex layer of the BEM, were computed using the OpenMEEG toolbox (Gramfort et al., 2010) which was also used later for computing the current density during source localization.

### Source localization-cloreta

Cortically Constrained Low Resolution Electromagnetic Tomography (cLORETA) (Pascual-Marqui et al., 1994) was used for source localization due to the fixed source-space it required for current localization, allowing for cross-participant ROI consistency. The source-space was the cortical BEM constructed in the previous section that accounted for the conduction of electric fields generated in the cortex and projected to the surface of the scalp where they were detected by EEG. The cortical constraint placed on the BEM was also necessary due to the inability of EEG to reliably resolve electrophysiological activity in subcortical regions of the brain. Source localization (procedure outlined in Figure 1E) was conducted independently for every epoch in each dataset in order to prevent regularization parameters and current activations from being computed by concatenating temporally non-contiguous epochs. The solution to the minimum-norm Lagrange multiplier problem for cLORETA is the Tikhonov regularized pseudoinverse solution for cortical current density expressed as follows:

(1) J=TΦ

(2) $$T={\left(K^{T}K\;+\lambda^{2}H^{T}H\;\right)}^{-1}K^{T}$$

where *J* is the current density on the cortex, Φ is the channel space data, ***T*** is the optimal transformation matrix from channel space to current density space, ***K*** is the discrete electrical lead field matrix, and ***H*** is the 2D spatial Laplacian of the cortical head model, introduced to spatially regularize the localized current on the head model grid points. λ is a regularization parameter initialized on a log-space, using generalized cross validation (GCV), and subsequently inferred using an iterative EM formulation derived from the Bayesian structure of the original current localization problem (Trujillo-Barreto et al., 2004). The cLORETA implementation in BCILAB (Delorme et al., 2010) was used for source localization.

Since the regularization parameter λ was recomputed for every epoch, a new transformation matrix (***T***) was also computed, leading to dynamic increases and decreases of spatial regularization in the final current solution as needed. This prevented both over-regularization in cases where current density ramped quickly between grid points, and under-regularization when channel space, and therefore current density magnitudes, were lower. A representative signal was extracted from each ROI by computing the signal power of the current localized at each gridpoint within a given ROI and selecting the gridpoint that exhibited the maximum power.

Based on this max-power selection heuristic, representative current signals were generated for the ROIs—a process which was applied to each of the 10 subjects individually, greatly simplifying network-level comparisons across test subjects since the nodes of the cortical network (the ROIs) were localized in the same location every time. This approach differs from traditional ROI determination methods involving ICs because the latter rely heavily on the common spatial localization of ICA outcomes, which is not guaranteed for multiple separate runs of ICA on different data, and, thus, may lead to inconsistencies for group level connectivity analysis (Thompson et al., 2011).

### Connectivity analysis–sift

Application of the maximum power heuristic on the results of cLORETA created a single time series that represented cortical current dynamics for each ROI selected through clustering. These time series representing ROIs (ROI signals) were used to construct linear (MVAR) models from which spectro-temporal causal interactions between ROIs were computed using the Source Information Flow Toolbox (SIFT) (procedure outlined in Figure 1F) (Delorme et al., 2011). Specifically, for each participant, the ROI signals were preprocessed by employing detrending and normalization across time and ensemble. The MVAR models were constructed by applying the Vieira-Morf lattice algorithm to the ROI signals, using a sliding window with a length of 550 ms and a window step size of 10 ms, leveraging the window size to decrease the number of the estimated MVAR coefficients (ratio of data points to parameters > 10:1)—a necessary provision arising from the lack of regularization in the Vieira-Morf algorithm. The optimal order for the MVAR model was determined automatically by averaging the optimal orders estimated by six different information criteria: Akaike Information Criterion, corrected Akaike Information Criterion, Hannan-Quinn Criterion, Schwarz-Bayes Criterion, Rissanen Prediction Error, and logarithm of Akaike's Final Prediction Error (Rissanen, 1978; Tu and Xu, 2012). Each of these information criteria was computed for the range of possible model orders between 1 and 30, and the optimal model order for each participant was automatically determined by identifying the minimum value for each of the six generated curves, computing the average, and rounding down to the nearest integer. The model order for the Lift and Reach/Saccade conditions were determined independently. After model order determination, tests of stability and consistency were performed for each MVAR model in order to validate the ability of the model to capture the dynamics of the ROI signal. The autocorrelation function and three different portmanteau tests were used to determine the whiteness of the model residuals. The percent consistency of the model was evaluated for every sliding window within the epoch, indicating, as a percentage, the relative amount of correlational structure within the data that had been captured by the model. A stability test was conducted on every window within the epoch the MVAR coefficient matrix was evaluated.

Pairwise causal relationships between ROIs were computed from the validated MVAR models by employing the (SdDTF) (Korzeniewska et al., 2008). The SdDTF connectivity $\eta_{ij}^{2}\left(f,t\right)\;$ at a given spectro-temporal point (*f, t*) between ROI-i and ROI-j is computed as follows:

(3) $$\eta_{j\to i}^{2}\left(f,t\right)=\frac{{\left|H_{ij}\left(f,t\right)\right|}^{\;}{\left|P_{ij}\left(f,t\right)\right|}^{\;}}{\sum_{f}\sum_{kl}{\left|H_{kl}\left(f,t\right)\right|}^{2}{\left|P_{kl}\left(f,t\right)\right|}^{2}}$$

where *H*_*mn*_ (*f, t*) is the MVAR transfer matrix between network nodes (ROIs) *m* and *n*, and *P*_*mn*_ (*f, t*) is the partial coherence of the signals for nodes *m* and *n*. The SdDTF connectivity metric is similar to conditional spectral Granger Causality, but only reports direct causal relationship between signals, removing spurious or indirect relationships caused by upstream network topology, particularly common inputs. Unlike Granger Causality, which utilizes AR model residuals to determine causal influence, SdDTF utilizes the AR transfer functions themselves (see Equation 3) allowing for straightforward generalization of the metric to multivariate systems, in this case a network with more than two cortical nodes. To control for the potentially large and unregularized AR transfer functions, DTF normalization is conducted across incoming and outgoing signals, and across frequencies. This normalized term is weighted by the partial coherence in order to incorporate the direct relationships between source nodes, thus mitigating the phantom source problem present in Granger causal metrics and yielding the full form of the direct Directed Transfer Function. This metric is further developed by evaluating it over a short sliding window across the epoch, an additional feature which serves to promote local stationarity of the ROI source signals and observe changes in connectivity dynamics between signals as the epoch progresses. The SdDTF measure was computed separately across epochs and frequencies of interest in this way, yielding a spectro-temporal asymmetrical causal connectivity matrix of SdDTFs with causal source nodes (FROM) defining the columns and sink nodes (TO) defining the rows of the connectivity matrix. Note that the diagonal elements of this matrix do not have relevant physical meaning for the purposes of this network analysis, and were, therefore, omitted.

A permutation-bootstrap approach was employed to determine the statistical significance of each SdDTF value within the asymmetric connectivity matrix (outlined in Figure 1G). The permutations were created by randomly mixing ROI-current density epochs from the control and test conditions with equal representation, generating an epoch ensemble on which connectivity analysis was conducted. The resulting ROI connectivity matrices from each permutation were accumulated to create a surrogate distribution. The values of each spectro-temporal position in this distribution were, then, sorted in ascending order, allowing for the associated *p*-value of the tested connectivity value to be computed in a non-parametric manner through its place in the ranked connectivity values at that spectro-temporal position of the surrogate distribution. This process was applied to both the Lift and Reach/Saccade conditions, where the null hypothesis (H_0_) was the lack of significant increase or decrease in connectivity between the evaluated condition and the case of randomly mixed ROIs. The alternative hypothesis (H_1_) was that the evaluated condition individually exhibited connectivity structure that was significantly different from the structure acquired by randomly intermixing the two conditions. The *p*-value for each spectro-temporal point was determined by using an averaged surrogate distribution computed across all 10 participants. A surrogate distribution for each of the 10 participants was computed by conducting connectivity analysis on 300 permutations of the original data, yielding 300 SdDTF values for each spectro-temporal position. The average surrogate distribution was computed by applying spectro-temporal position-wise averages across all 10 surrogate distributions for each of the 300 values and, subsequently, sorting in ascending order the resulting average values at each spectro-temporal position. With 300 permutations computed for each subject, there was sufficient *p*-value resolution to establish which time-frequency points passed the α = 0.05 threshold. This threshold was automatically adjusted since the statistical significance of each spectro-temporal point was evaluated independently by applying the False Detection Rate (FDR) control method as a means of accounting for the multiple comparisons problem (Benjamini and Hochberg, 1995).

The difference between statistically significant values of average connectivity matrices corresponding to the Lift condition and Reach/Saccade condition was computed in order to determine the statistically significant changes in the cortical network between the two conditions.

## Results

### IC clustering outcome

The ROIs selected for source localization based on the cluster centroids were: bilateral (left/right) inferior occipital (Occ), superior parietal (Par), and precentral gyrus motor (Mot) cortices, as well as, anterior cingulate cortex (ACC), supplementary motor area (SMA), and precuneus, whose left and right hemisphere grid point regions were combined and treated as a single anatomical region during source localization and ROI collapse.

**Figure 3:**
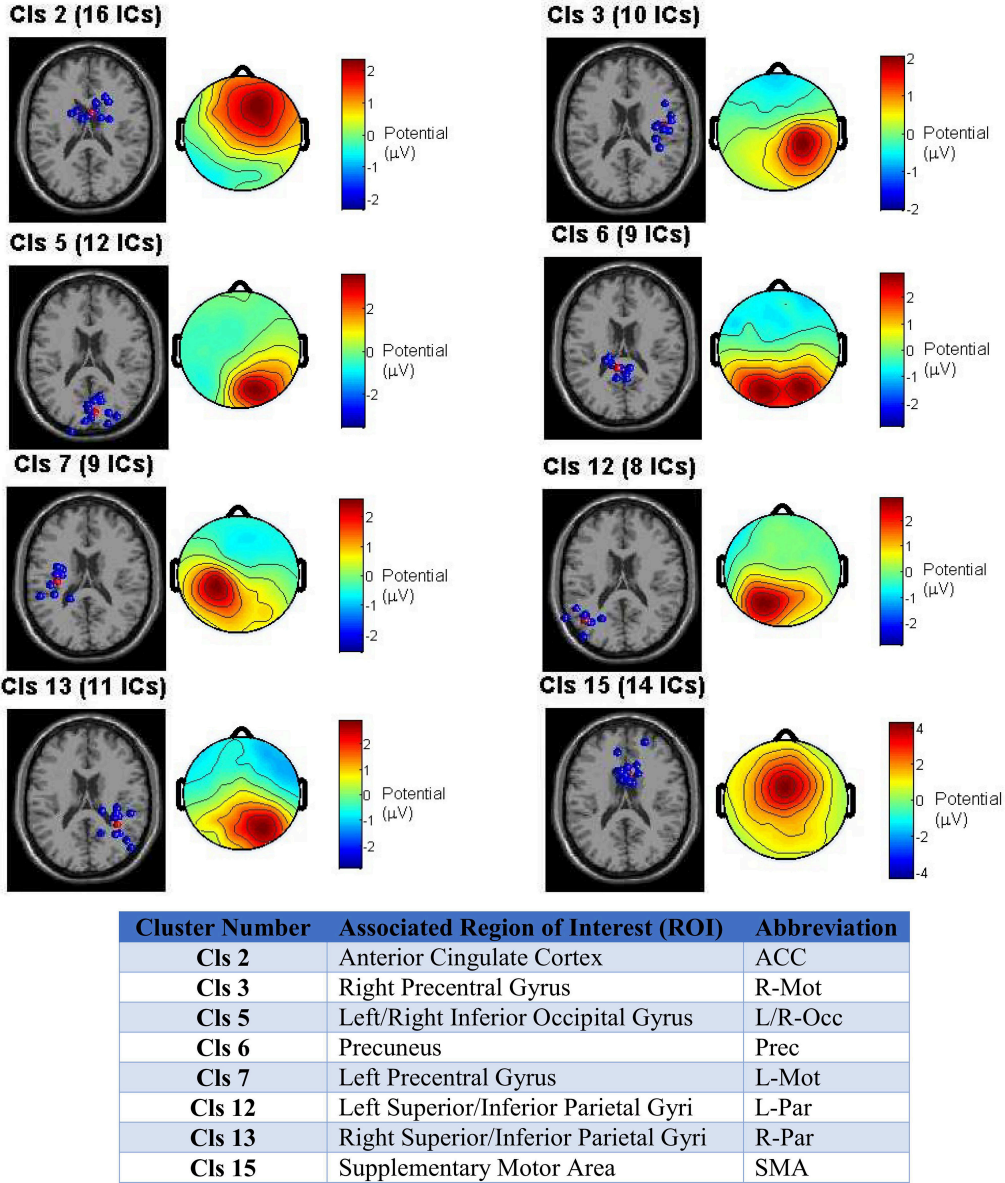
**Clustering outcome across all test subjects for the Reach/Saccade condition**. IC dipole locations in each cluster are displayed in blue on a standard MRI volume, with the cluster centroid in red (centroid itself is not an IC, interpolated from other dipoles in cluster). The scalp maps are composites displaying the average electric potential on the scalp arising from the equivalent IC-dipoles within each cluster. The cluster centroids provide the foundation for subsequent ROI selection by association with the anatomical structure(s) the cluster occupies. The associated ROIs for each cluster are shown in table y. Note that for non-bilaterally separated ROIs, the areas in the left and right hemispheres were combined due to close proximity across the medial longitudinal fissure.

Figure 3 depicts the clustering outcome for the reach/saccade condition, showing the dipole cluster and average scalp projection for the eight clusters from which ROIs were determined. The 3D dipole maps plotted on a standard MNI MRI volume demonstrate the spatial tightness of the clusters selected as candidates for ROIs, while the average composite topographical maps indicate that there is consistency in both the orientation and di-polarity of the ICs in each cluster. All of the selected clusters feature a relatively tight positive spike in electric potential over the cortical area where the equivalent dipoles are placed; suggesting that the collection of dipoles within the cluster originate from similarly oriented pyramidal cells in gray matter, generating electrical dipoles whose resultant field produces the positive potential present in the scalp maps. Were the IC clusters not as spatially tight, or comprised of dipoles oriented in different directions, the average scalp maps would have electric potential much lower in magnitude and dispersed over a much wider area of the brain. The spatial tightness of the dipoles and the scalp maps suggests that a robust clustering outcome was achieved, and the associated ROIs were reliably utilized with this data in subsequent analysis.

The determination of ROIs from ICs is not necessarily a one-to-one mapping. A single cluster can provide evidence for inclusion of multiple ROIs, as is the case with cluster 5, for example, whose dipole locations span across both hemispheres. So, when identified with the Talairach atlas, the ROIs fall in bilateral regions of the Occipital Cortex (Inferior gyri) that are too far from each other to be grouped across the medial longitudinal fissure, as was the case with all of the other midline ROIs.

**Figure 4:**
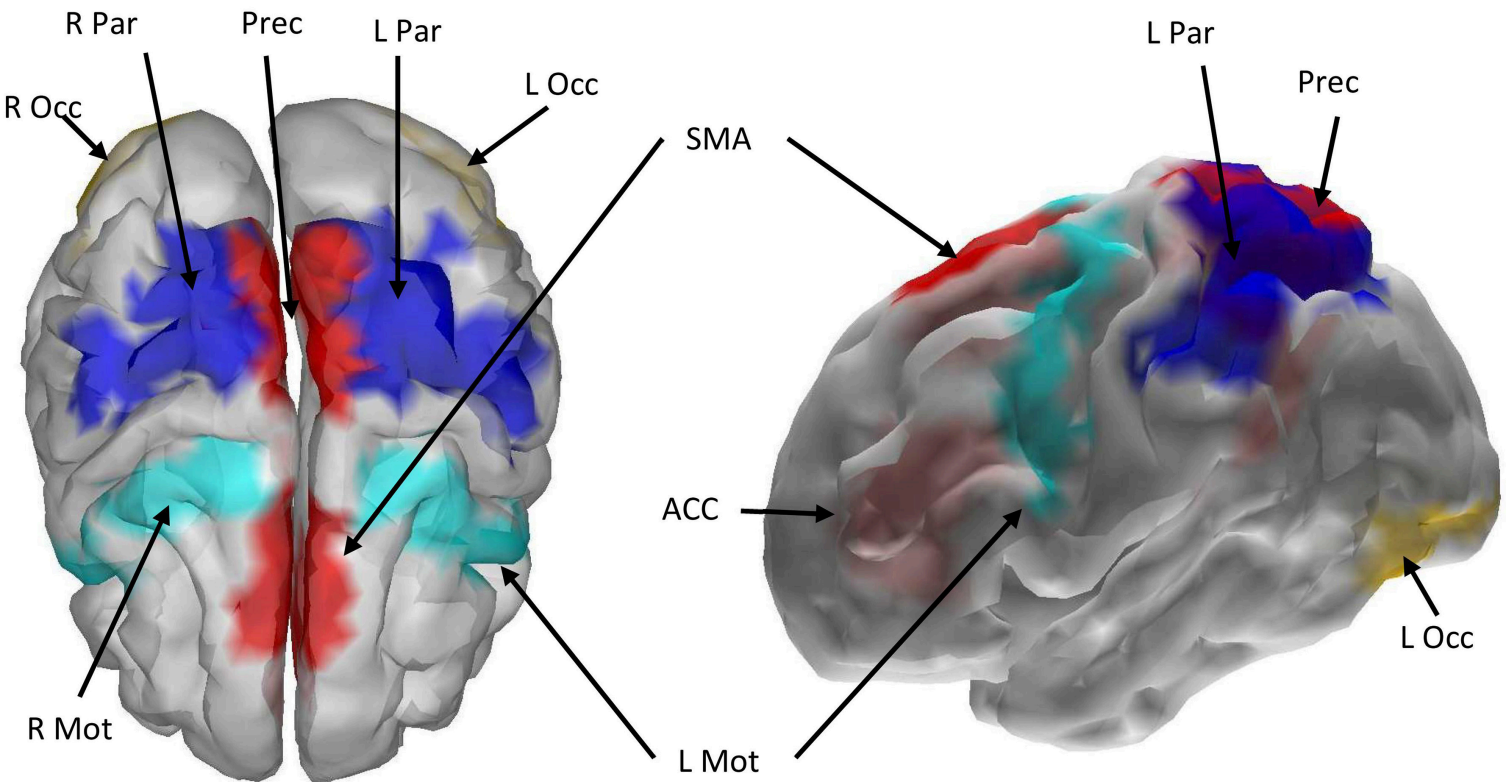
**Cortical boundary element head model with the nine selected ROIs marked with color**. Red color indicates that the left and right hemispheres of the ROI were merged and treated as a single ROI during source localization and signal extraction. Other colors indicate that bilateral cortices were selected across the hemispheres. ROI abbreviations are: Mot, Precentral Gyrus; ACC, Anterior Cingulate Cortex; SMA, Supplementary Motor Area; Prec, Precuneus; Occ, Inferior Occipital Gyrus; Par, Superior and Inferior Parietal Gyri.

All nine ROIs selected for the cortical network are displayed in Figure 4, with color-coded grid-points associated with each ROI as follows: midline ROIs are colored Red (ACC, SMA, Precuneus), and bilaterally (left and right) situated ROIs away from the midline (Mot, Par, and Occ) are dark blue, light blue, and yellow respectively.

### MVAR model construction and validation

**Figure 5:**
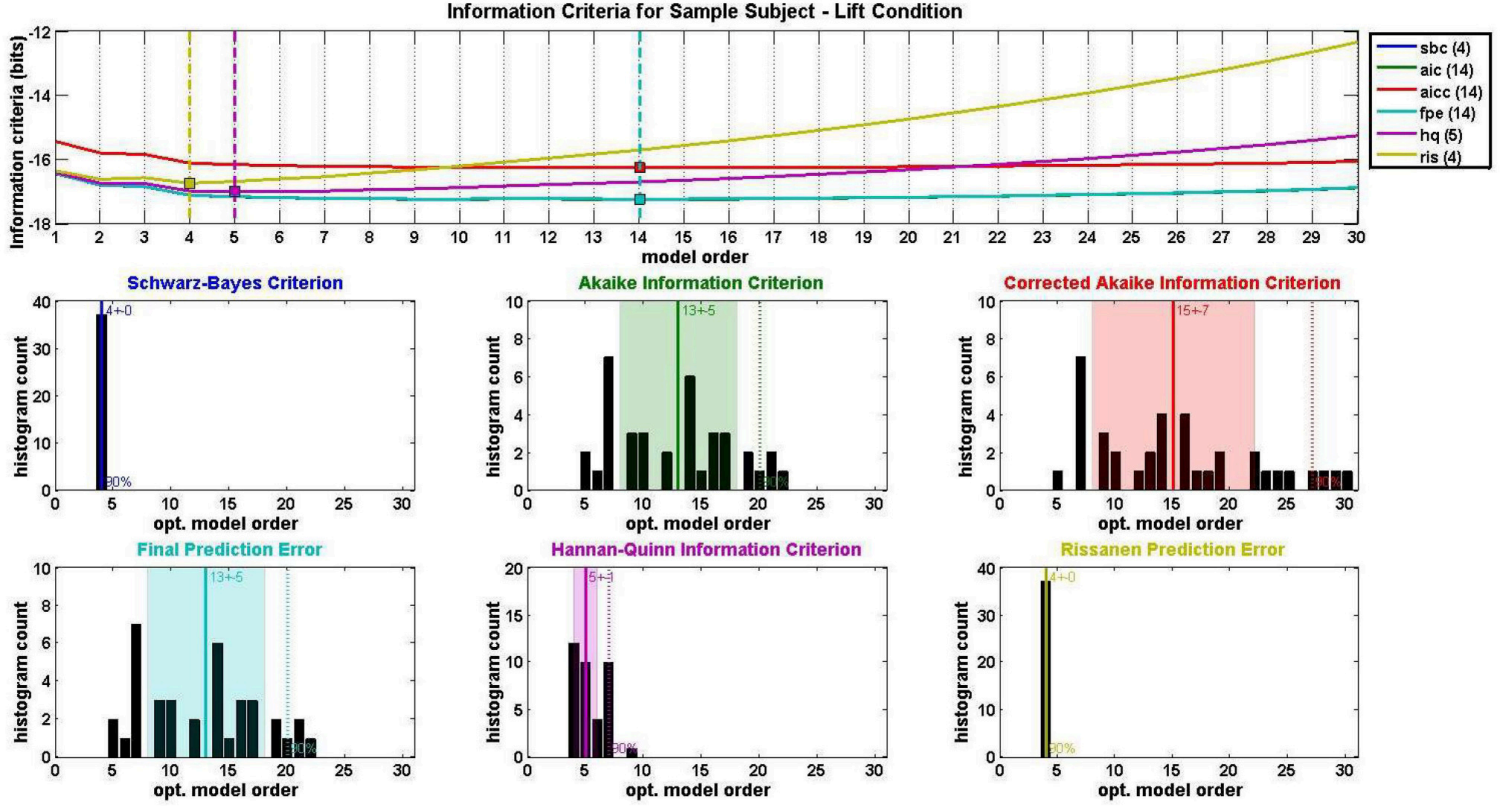
**MVAR model order selection for the lift condition of a single test subject**. Each of six information criteria (Schwarz-Bayes Criterion, Akaike Information Criterion, Corrected Akaike Information Criterion, Final Prediction Error, Hannan-Quinn Information Criterion, and Rissanen Prediction Error Rissanen, 1978; Tu and Xu, 2012) were evaluated for model orders 1–30 and averaged across all condition trials. Optimal model order corresponds to the minimum of each information criterion curve. Distributions of optimal model order for all trials are shown in histograms for each of the six information criteria used. Center lines and shaded regions in each histogram indicate the mean and standard deviation respectively for each information criterion.

Model order selection was conducted with an average MVAR parameter-to-datapoint ratio of 0.096 for the Lift condition and 0.034 for the Reach/Saccade condition. The window size was selected so that, in all cases, this ratio never exceeded 0.1, as is recommended for a well-posed MVAR model computed via the Vieira-Morf Lattice Algorithm. For the Lift condition, the average model order across all subjects was 7.4 with a standard deviation of 1.35, and for the Reach/Saccade condition, the model order average was 7.2 with a standard deviation of 1.03. Figure 5 shows the output of the model order selection criteria pertaining to the MVAR model order of the Lift condition for a single participant. The top graph shows the average (across epochs and sliding windows) value of each criterion over model orders from 1 to 30. The histograms that follow show the order distribution using each of the criteria.

**Figure 6:**
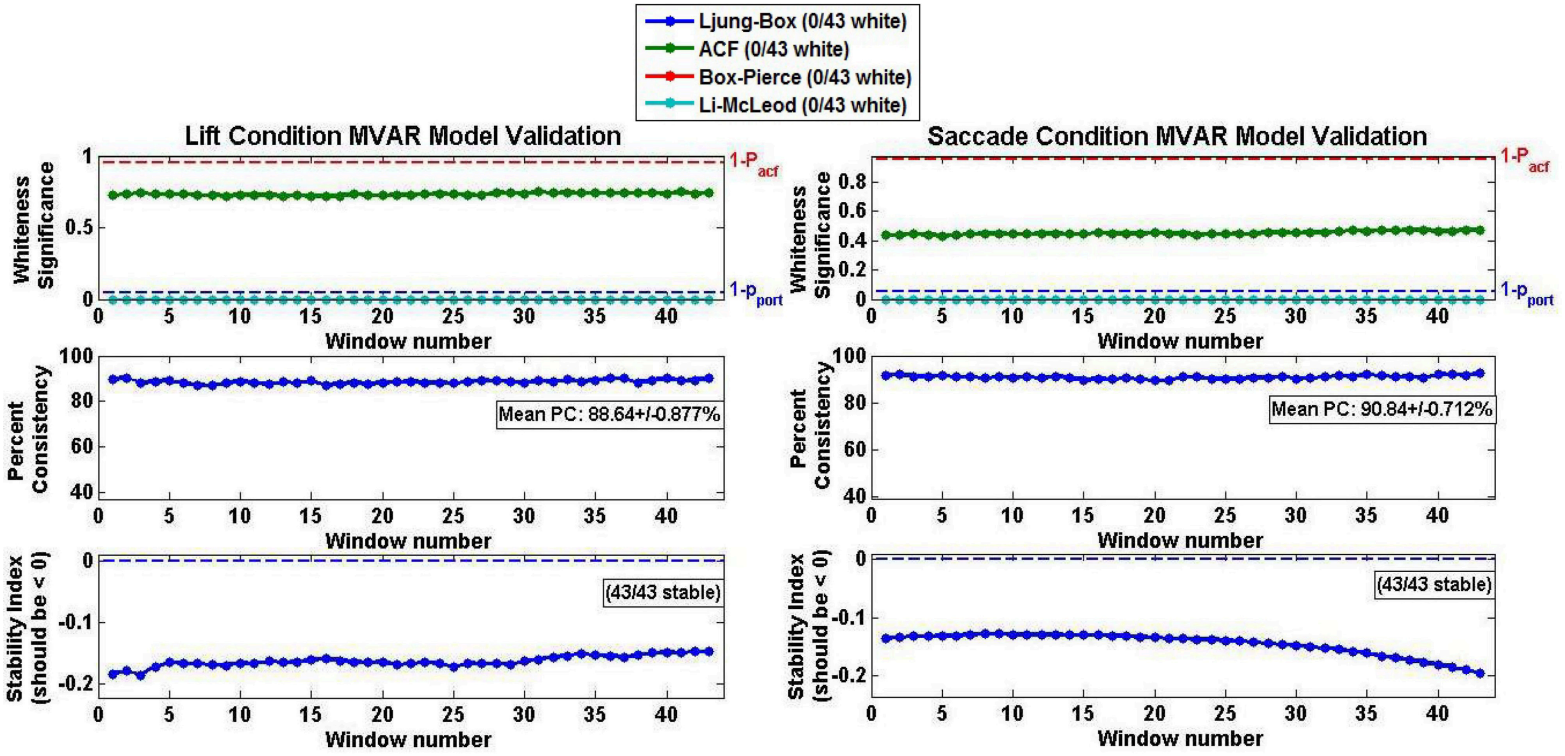
**Model validation criteria for Lift (left) and Reach/Saccade (right) Conditions averaged across all 10 subjects**. Whiteness of AR model residuals (top row) evaluated using Autocorrelation function (ACF) and three different Portmanteau tests. None of the MVAR models passed any of the whiteness tests, suggesting that none of the models captured adequately the modeled temporal dynamics. Percent consistency (middle row) indicates percent of correlational structure of original data captured by the model. Percent consistency was uniformly high as its values were in the order of 90% with small standard deviations. Stability index (bottom row) is log of largest eigenvalue of AR model. Negative index implies that largest eigenvalue is less that one, and thus, the MVAR models were stable. Note, that all tests were conducted individually for every sliding window (43 in total) across the epoch, hence the 43 data points in each graph.

The MVAR model validation results averaged across all subjects and both conditions are shown in Figure 6. The average model performance was highly representative of the individual performance of each subject's MVAR model since, in all cases, the MVAR model was stable across the entirety of the epoch, as indicated by the natural logarithm of the largest eigenvalue being negative (Figure 6 Stability Panel). The percent consistency for both Lift and Reach/Saccade conditions was above 90% and the standard deviation in the percent consistency was <1.0% (0.877% for Lift and 0.712% for Reach/Saccade), indicating that the high consistency of the MVAR model with respect to the data was maintained for the duration of the epoch (all 43 sliding windows within each epoch). None of the computed MVAR models passed the residual-whiteness tests at any point during the epoch, suggesting that there was statistically significant correlational structure exhibited by the MVAR model residuals, and that the MVAR modeling did not succeed in capturing the entirety of the temporal dynamics exhibited by the network nodes during the epoch.

### Spectro-temporal connectivity

**Figure 7:**
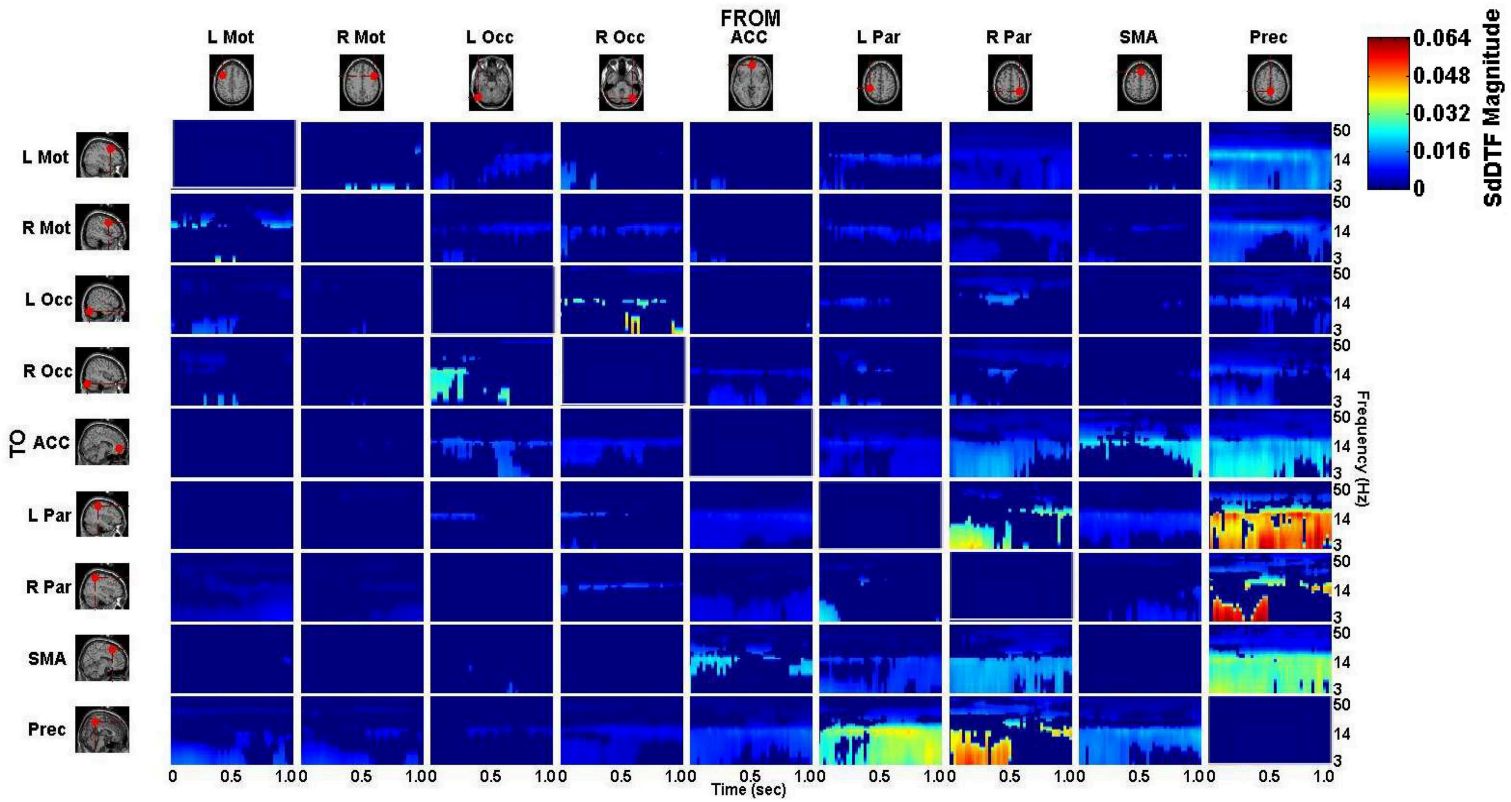
***p*-value thresholded spectro-temporal asymmetric cortical connectivity matrix for the lift condition**. Network sources are tabulated at the columns and sinks at the rows. The dynamic causal relationship between each source and sink is expressed by the corresponding SdDTF. Non-zero connectivity is displayed only at spectro-temporal points with *p* < 0.05, thus only displaying statistically significant connectivity in the cortical network for the Lift condition. Note the frequency axis is plotted on a logarithmic scale to more clearly show lower frequency band connectivity, where the majority of causal flow occurs in this network.

**Figure 8:**
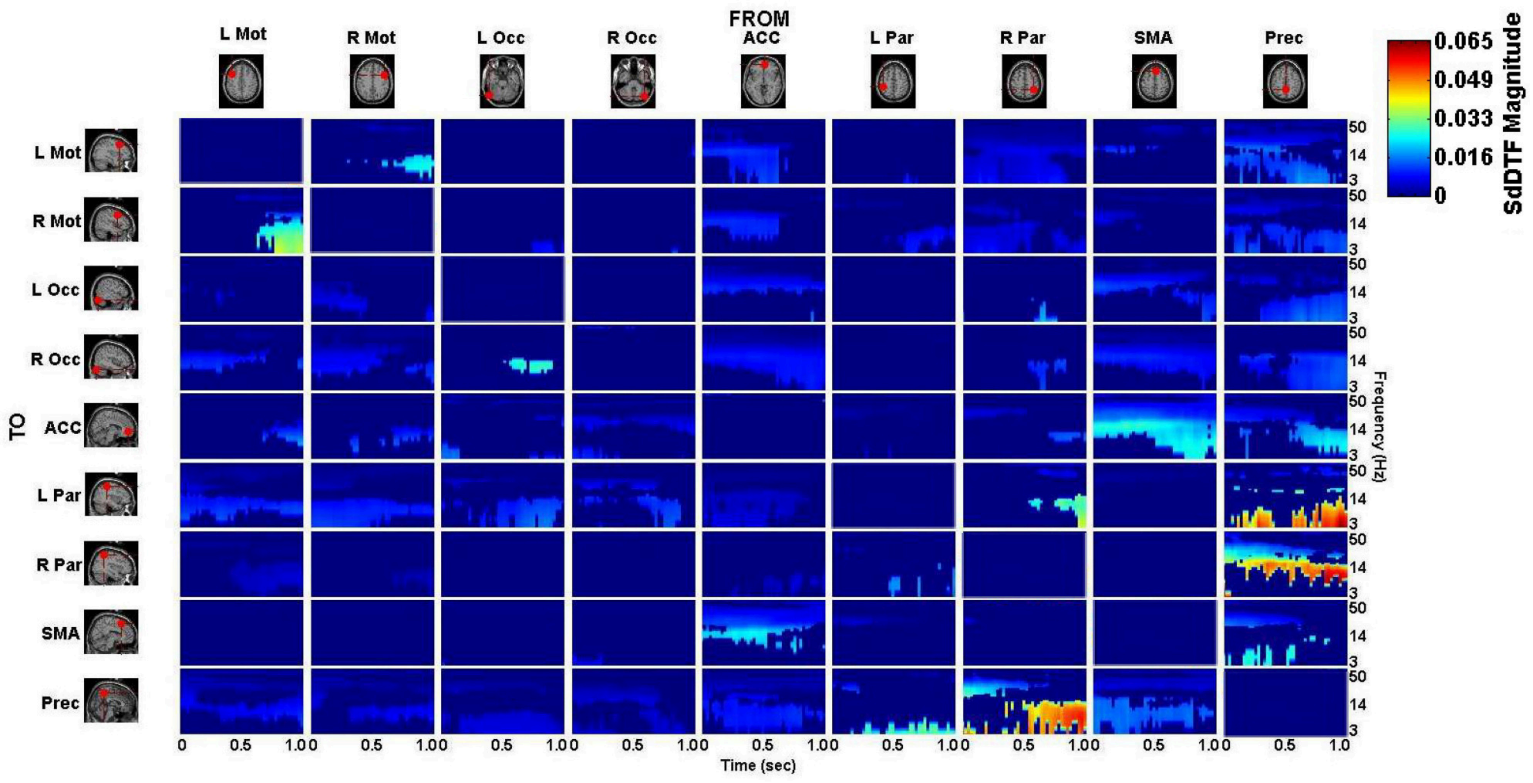
***p*-value thresholded spectro-temporal asymmetric cortical connectivity matrix for the reach/saccade condition**. Network sources are tabulated at the columns and sinks at the rows. The dynamic causal relationship between each source and sink is expressed by the corresponding SdDTF in the pertinent location in the connectivity matrix. Non-zero connectivity is displayed only at spectro-temporal points with *p* < 0.05, thus only displaying statistically significant connectivity in the cortical network for the Reach/Saccade condition. Note the frequency axis is plotted on a logarithmic scale to more clearly show lower frequency band connectivity, where the majority of causal flow occurs in this network.

The connectivity computed between the ROIs in the defined cortical network is displayed in Figure 7 for the lift condition and Figure 8 for the reach/saccade condition, with statistically significant spectro-temporal connectivity (*p* < 0.05) thresholded for each matrix respectively.

**Figure 9:**
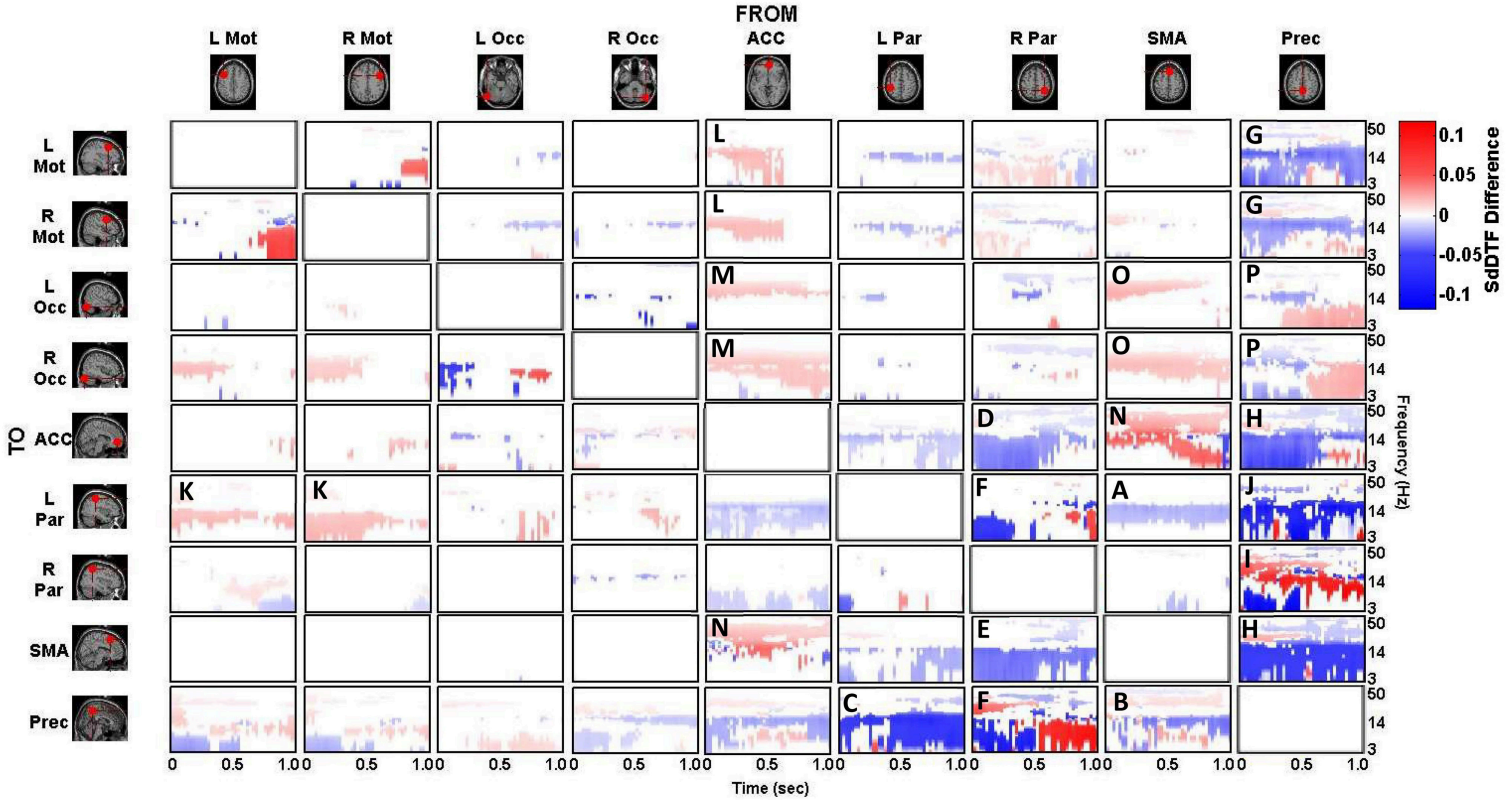
**Difference of statistically significant values (*p* < 0.05) of the connectivity matrix between the Reach/Saccade and Lift condition**. Warmer colors indicate Reach/Saccade > Lift, cooler colors indicate Lift > Reach/Saccade. Note, that during Reach/Saccade, information flow is facilitated from both precentral gyri (L/R Mot) to left parietal area **(K)** and ACC exhibits greater influence on precentral gyri during planning (0–0.5 s) as well as the visual cortices (L/R Occ) during both planning and execution **(L,M)**; during Lift, Precuneus is much more active both inflow **(B,C,F)** and outflow **(G,H,J,P)** except when communicating with the right parietal area **(I)**, especially during the execution phase (0.5–1.0 ms) of the task. The SMA also drives activity in the visual cortices **(O)**, as well as displaying outflow to the ACC **(N)** and L Par **(A)** during Reach/Saccade and Lift respectively. Also during Reach/Saccade, the parietal region ipsilateral to the moving arm (R Par) demonstrates reduced outflow to both ACC and SMA **(D,E)** relative to Lift. Brighter red and brighter blue show stronger Reach/Saccade and Lift connectivity respectively.

The difference between the *p*-value thresholded connectivity matrices for Lift and Reach/Saccade, shown in Figures 7, 8 respectively, was computed and shown in Figure 9. Since the lift (control) condition was subtracted from the reach/saccade (test) condition, the warmer (brighter red) regions of the matrix indicate that connectivity was stronger during the reach/saccade and the cooler (brighter blue) regions indicate that connectivity was stronger during the lift.

The majority of lift-dominant causal interactions originate from the supplementary motor area, parietal areas and the Precuneus. During Lift, the SMA exhibits notable outflow to the Left Parietal Area (L Par) and the Precuneus. The SMA drives activity in the L Par over a constant frequency range (8–15 Hz) during both planning and execution (Figure 9A). However, a dichotomy between planning and execution exists in the connection form the SMA to the Precuneus, with sporadic low-frequency connectivity (3–14 Hz) present during planning that narrows in frequency band (8–14 Hz) and becomes more consistent during execution (Figure 9B). From L Par, the only significant outflow of information occurs to the Precuneus, dominating lower frequencies (3–14 Hz) during part of the early planning phase and the entirety of the execution phase of the lift (Figure 9C). From R Par, information flows to the ACC across low frequencies (3–14 Hz) during the planning phase and for some of the execution phase, though the causal connection is limited to a narrow frequency band (10–14 Hz) by the end of the epoch (Figure 9D). Similar connectivity is present with the SMA, although in the case of information flow from R Par to SMA, the connectivity does not subside as late in the execution phase as in the case with the ACC (Figure 9E). The R Par exhibits another connectivity motif, providing strong causal influence on both the Left Parietal Area and the Precuneus during the planning phase of the lift over a low frequency band (3–10 Hz), and with relatively higher power compared to its other causal influences (Figure 9F). The Precuneus exhibits the greatest amount of causal outflow during lift, acting as an information source for the majority of the other ROIs in the network. The Precuneus has relatively lower-power (lighter blue shading) influence on both precentral gyri, during both planning and execution, with the band of active connectivity narrowing when transitioning from planning to execution (Figure 9G). The Precuneus sends information with a similar spatiotemporal structure to the ACC, but with slightly greater causal power, hence the brighter shading of the connectivity during the planning period (Figure 9H). The influence the Precuneus has on the SMA is stronger still (even darker shade of blue), and now occupies the entire duration of the epoch and maintains signal bandwidth (3–14 Hz) (Figure 9H). The Precuneus influences both parietal areas very strongly during lift, with R Par receiving information only during the planning phase across a low frequency band (3–10 Hz) (Figure 9I), and L Par receiving information across a slightly larger band (3–14 Hz) and for both planning and execution. During execution, however, the connectivity is limited to the higher end of that frequency band (10–14 Hz) (Figure 9J).

Within reach/saccade-dominant activity regions, which are defined by the red spectro-temporal regions of Figure 9, greater connectivity is present in the frontal regions of the cortex. Specifically, both the left and right precentral gyri exhibit stronger connectivity to the left parietal composite area during the planning phase of the epoch across lower frequencies (3–14 Hz), with this connectivity decreasing in power relative to lift during the execution phase (Figure 9K). Additionally, the anterior cingulate cortex (ACC) exhibits increased outflow of information across lower frequencies (3–14 Hz) to both precentral gyri again during the planning phase (Figure 9L). The ACC also appears to feed information to the left and right visual cortices across a narrower frequency band (8–14 Hz) during the planning phase (Figure 9M), with the bandwidth of information flow encompassing lower frequencies from ACC to R Occ as the epoch progresses from planning to execution. For the reach/saccade condition, the SMA exhibits a greater amount of causal influence on the ACC than any other cortical node, with a strong 14–40 Hz communication band present during the planning phase that dips down to lower frequencies (3–8 Hz) as the subject transitions from planning to execution (Figure 9N). The ACC reciprocates some of this activity to the SMA, with higher frequency (15–40 Hz) communication during the planning phase that dies down during the execution phase (Figure 9N). The SMA also exhibits some information outflow to the visual cortices, with the L Occ flow subsiding by the end of the planning period, and the R Occ flow remaining constant in both frequency band and relative amplitude over the entire epoch (Figure 9O). The right parietal composite area (R Par) exhibits causal influence on the Precuneus in higher frequencies (20–25 Hz) during the planning phase, and significantly stronger lower frequency (3–13 Hz) influence during reach/saccade execution in comparison to the lift execution (Figure 9F). The last of the significant communication motif that is strong during the reach/saccade conditions originates from the Precuneus. The influence of Prec on L/R Occ is most prevalent during the execution phase (Figure 9P). In both cases, however, the precuneus predominantly drives lower frequency activity (3–14 Hz), with a significant decrease in connectivity magnitude above 14 Hz. There is also evidence of temporally localized higher power bursts of connectivity across low frequency from the Precuneus to the right parietal area, beginning early in the planning period in the 14–20 Hz band and terminating at the end of the epoch after drifting down to 8–13 Hz (Figure 9I).

## Discussion

### Neurophysiological connectivity

A cortical network associated with the Lift and the Reach/Saccade conditions was identified across 10 subjects, involving distinct ROIs, and the dynamic causal interactions between pairs of ROIs were computed for each condition. The cortical network, the ROIs, and the dynamic causal connections between pairs of ROIs exhibited physiological plausibility consistent with previous results reported in the literature, although some important differences were noted as outlined further below. The bilateral Inferior Occipital Gyri coupled with adjacent Occipital regions, known to form the Visual ROIs, represent primary processing of the visual stimuli present in the reaching task (Blumberg and Kreiman, 2010). The various motor regions, including bilateral Precentral Gyri (housing the Left and Right Motor Cortices) and the bilaterally grouped Supplementary Motor Area, have been implicated in the planning and execution of the physical movement, e.g., in this case of the participant's arm to reach the presented target as well as the saccade of the eyes to focus on the target (Johnson et al., 1996; Nachev et al., 2008; Wang et al., 2010). Parietal activity is largely implicated by existing literature in guiding action directed at visual targets (Blangero et al., 2009; Filimon, 2010). The Anterior Cingulate Cortex, a grouped ROI consisting of the Left and Right cortices merged across the longitudinal fissure, is thought to be involved in higher level decision making and, of particular interest in this case, the translation of intentions to actions (Kennerley et al., 2006). The Precuneus, also bilaterally grouped across the longitudinal fissure, has been shown to be directly involved in both visual and non-visual reaching tasks in both macaque monkeys and humans, being responsible for the processing of both visual and proprioceptive sensory information during the task (Filimon et al., 2009).

Though entirely possible to treat the three grouped ROIs (SMA, Prec, and ACC) as distinct, the close proximity of the bilateral ROIs to each other within the longitudinal fissure and the spatially-regularized source localization can lead to significant overlap of posterior current density between the ROIs, generating spurious causal interhemispheric coupling, which is avoided by considering those three ROIs to be trans-hemispheric.

Inspection of the connectivity during the lift and reach/saccade condition, shown in Figure 9, reveals that the cortical network exhibits relatively lower strength, but widespread effective connectivity during the reach/saccade condition, and more focal, higher-strength connectivity during the lift condition. In the case of the reach/saccade condition, the ACC exemplifies this widespread, low-power connectivity profile, driving activity in both Left and Right Precentral Gyri during the planning period, and driving activity in both visual cortices for both planning and execution of the reach. The connection from the ACC to L/R Mot (Figure 9L) suggests a causal influence of a decision making center in the frontal cortex onto regions directly involved in the planning and execution of a reach and saccade, a connectivity motif that has been observed before using other neuroimaging modalities (Asemi et al., 2015). Furthermore, this causal influence is shown only to exceed lift-connectivity magnitude during the planning period, indicated by the lack of ACC to L/R Motor interaction after 0.5 s, suggesting that after the movement is planned, execution no longer requires direct influence of the ACC onto the Motor cortices. On the other hand, the ACC to L/R Visual connections (Figure 9M) remain elevated for the entirety of the epoch during the reach/saccade condition, indicating a possible involvement of the cognitive-control and error detection functions of the ACC (Stevens et al., 2011) in visual processing during execution. The task-positive connections formed by the ACC exhibit higher frequency connectivity (Figures 9 L–N), with the lower end of these connectivity bands lying in the α (8–13 Hz) wave range, which is expected considering the motor-relevance of the α-band to indicate inactivation of motor regions (Brinkman et al., 2014), and the higher end of these connectivity bands stretching into the 30–50 Hz range, consistent with the generally higher-frequency content of cognitive cortical processes (Buzsáki and Silva, 2012).

However, this does not seem to be the case for the information flow from the SMA to the ACC (Figure 9N), wherein a high α/low β band connection (10–20 Hz) present during the planning period migrates down to lower frequencies (3–8 Hz), occupying the θ band by the end of execution. A possible explanation for this exception stems from the suggested involvement of the SMA in the real-time control of actions during execution, by which it might provide feedback to the ACC regarding the accuracy of the conducted reach (Nachev et al., 2008). The connections from L and R Mot to L Par (Figure 9K) exhibit a connectivity profile similar to the ACC → L/R Mot interaction, with lower-power, reach/saccade-dominant connectivity during planning that is absent during execution. While both the composite parietal regions and the precentral gyri have been implicated in visuo-spatial processing and reaching to identified targets, the analysis indicates that their interaction is limited during the planning phase of a reach, and is not required in order for the reach to be executed after planning. A strong, sharply divided connection is also observed from the Right Composite Parietal Region to the Precuneus (Figure 9F)—a connection which is facilitated during lift-planning and reach/saccade-execution, and suppressed during reach/saccade-planning (for low frequencies) and lift-execution. The connectivity profile suggests that during reach/saccade planning, R Par predominantly communicates with the Precuneus through higher frequencies (14–30 Hz), which is of great significance to the Precuneus since it normally communicates with other nodes in the Default Mode Network through this frequency band (Neuner et al., 2014). The transition to lower frequency connectivity (3–14 Hz) during Reach/Saccade execution may simply be a feature of task demands, since similar communication was seen in Figures 9H,I,M,N,P, suggesting a generalized tendency for visuo-spatial and motor related cortical regions to communicate through lower frequency bands when the reach/saccade is being executed. The reach/saccade-dominant dynamics of the connection from the Precuneus to R Par (Figure 9I) also exhibit characteristics similar to the other motor regions, where the high-frequency planning connectivity gives way to lower frequency execution connectivity. However, during the epoch, the frequency range of the connectivity varies periodically, dipping to lower frequencies and, subsequently, returning to higher frequencies (Figure 9I, red profile) at the rate of 5 Hz. This behavior suggests that the Precuneus dynamically alters the frequencies over which it communicates with R Par in a periodic manner. The persistence of this behavior through both planning and execution suggests that the behavior is associated with generalized motor-reach information processing, though the decrease (Figure 9I, red profile) at the transition between planning and execution at 0.5 s supports the involvement of this periodicity in the motor task being executed by the subject.

**Figure 10:**
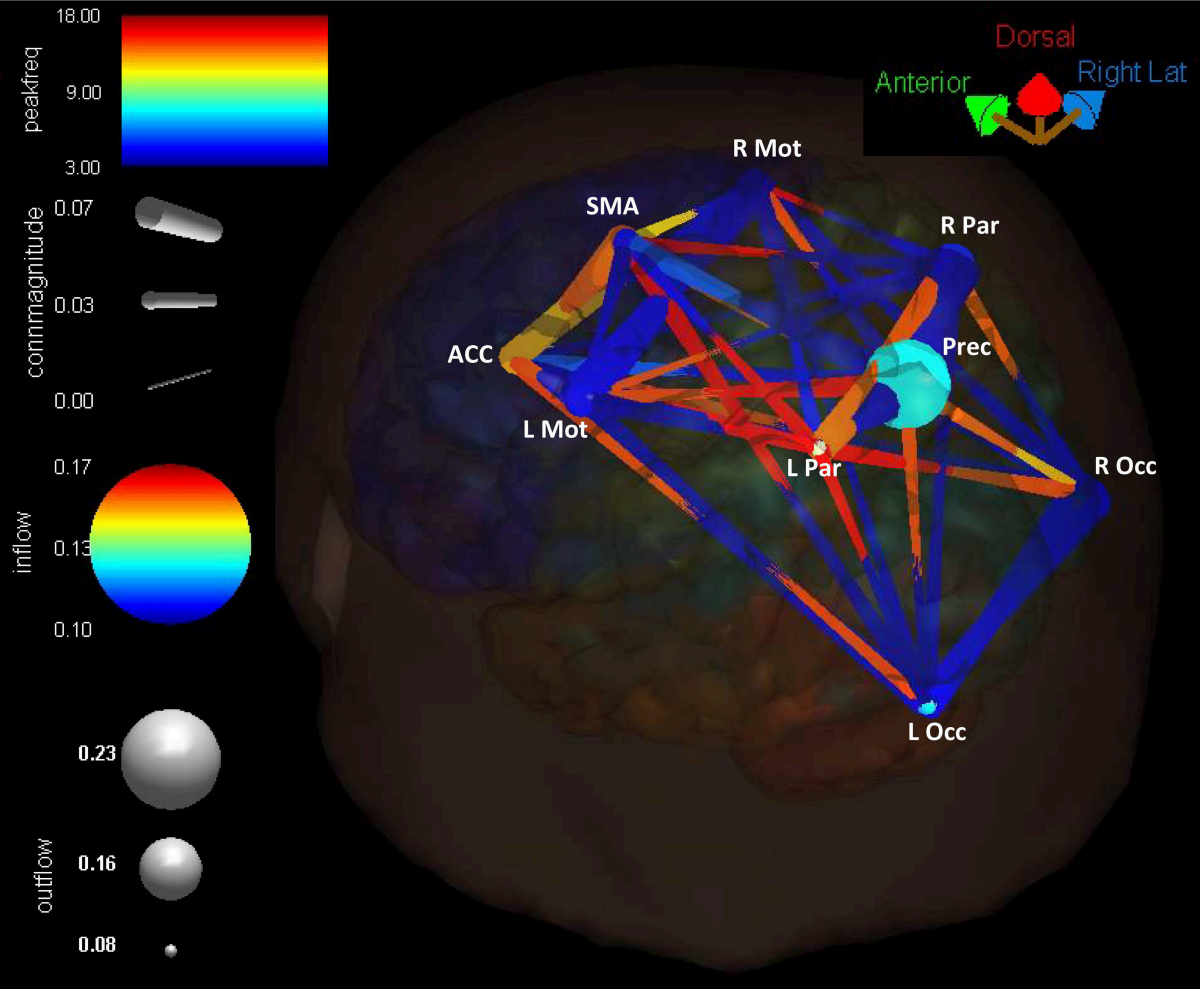
**Cortical connectivity present during the planning phase of the Reach/Saccade condition, shown in a 3D (BrainMovie3D) rendering of a cortical volume (Delorme et al., 2011)**. ROIs are represented by labeled spheres of varying size and color, with the magnitude of causal information in-flow indicated by color and the magnitude of causal information out-flow indicated by size. Connections between ROIs are represented by tapered cylinders with varying color and diameter. Cylinder tapering indicates the direction of information flow, cylinder width indicates flow magnitude, and cylinder color represents the dominant frequency of communication, with cooler colors for lower frequencies and warmer colors for higher frequencies. Causal information flow was integrated across all computed frequencies (2–50 Hz), and is shown here for *t* = 250 ms, during the planning period. The 3D rendering demonstrates the dominant role of the Precuneus as an information source, indicated by its large size, the higher power/frequency coupling of frontal regions (ACC, SMA, L/R Mot) indicated by warmer colored and larger connecting cylinders, and higher frequency communication present in the network during reach planning.

Connectivity present in the cortical network is summarized in Figure 10, where the Reach/Saccade condition is shown in a 3D (BrainMovie3D) rendering of a cortical volume (Delorme et al., 2011). The ROIs are represented by labeled spheres of varying size and color, with the magnitude of causal information in-flow indicated by color and the magnitude of causal information out-flow indicated by size. Larger size spheres indicate larger information out-flow and warmer colored spheres (brighter red) indicate larger information in-flow. Connections between ROIs are represented by tapered cylinders with varying color and diameter. Cylinder tapering indicates the direction of information flow, while cylinder width indicates flow magnitude, and cylinder color represents the dominant frequency of communication, with cooler colors for lower frequencies and warmer colors for higher frequencies. Causal information flow is integrated across all computed frequencies (2–50 Hz), and is shown here (Figure 10) for *t* = 250 ms, that is, during the planning period. The 3D rendering demonstrates the dominant role of the Precuneus as an information source, indicated by its large size, the higher power/frequency coupling of frontal regions (ACC, SMA, L/R Mot), indicated by warmer colored and larger connecting cylinders, and the higher frequency communication present in the network during reach planning.

### Framework limitations

The above discussion of neurophysiologically relevant findings serves to demonstrate the nature of the conclusions that can be drawn through the application of this algorithmic framework. Application of this method yields findings that are both consistent with existing literature, and reveal connectivity information in the cortex with an immense degree of detail, allowing for the documentation of source-space spectro-temporal information flow. There are, however, limitations with the current approach which must be considered.

The source orientation assumptions applied to the cortical BEM for the source space held that all cortical dipoles were oriented normally to the surface of the BEM, a canonical assumption that has been previously employed for BEM construction (Fuchs et al., 2002), and is motivated by the perpendicular orientation of cortical pyramidal cells to the cortical surface. Applying this assumption simplifies solving the inverse problem by reducing the number of variables to be estimated to *N*_*g*_ (number of BEM grid-points) from 3^*^N_*g*_, as is the case when accounting for the *x, y*, and *z* components of the current at each grid-point. However, this assumption greatly restricts the form of the computed current density, and does not allow for any data-driven flexibility with the computed dipoles. Utilizing the position-free BEM and associated Lead Field Matrix would allow for much greater flexibility, allowing for cortical dipole orientation to be computed either jointly with the current density, or after source localization using the current density, and thus identifying orientations through observed data instead of through a prior model.

Another potential drawback of the current source-localization algorithm is the Laplacian regularization utilized by cLORETA. The smoothing features of the discrete Laplacian when used as a Tikhonov matrix cause the estimated current density to be blurred over large regions of the cortical surface. This blurring can cause current density from a single source to spill over into multiple adjacent ROIs and potentially skew causal interactions between these ROIs in a manner that is not corrected by using the SdDTF connectivity metric. Preliminary investigations using this data suggest that this spill-over does not have a significant effect on causal analysis in this proposed framework, but further investigation would be prudent to ensure that the computed connectivity dynamics are not skewed by the choice of cLORETA as the source localization algorithm.

The validity of the presented connectivity dynamics is supported by the statistical analysis of the SdDTF connectivity matrix and the validation conducted on the fitted MVAR models. Though a large amount of the computed connectivity was statistically significant, the residuals of the computed MVAR models failed the whiteness tests because not all correlational structure of the localized current was captured by the models, suggesting the existence of non-linear dynamics. These non-linear dynamics are readily visible in the model prediction error and suggest that non-linear AR modeling is required to effectively capture these dynamics in the MVAR coefficients. However, all models exhibited a large Percent Consistency in the order of 90%, indicating that a very large percentage of the correlational structure of the localized current in each node was captured by the MVAR. The high percent consistency of the model indicates that these nonlinearities constitute a small portion of the localized current, and the computed MVAR models are able to account for the vast majority of the observed dynamics.

### Extensions to real-time BCI applications

In spite of the potential limitations discussed in the previous section, the proposed algorithmic framework has great potential as a means for extracting connectivity features in real time that can be used for brain state identification and BCI control. While in its current form, the framework is designed for off-line processing, Mullen et. al have demonstrated in Mullen et al. (2015) that elements of this framework can be applied in a real-time BCI setting and, with high accuracy, decode brain states using the spectro-temporal connectivity features computed through the use of this framework. The linear nature of the inverse problem formulated through cLORETA allows for rapid localization of current density in the cortex by application of the closed-form solution given in equation 2. Furthermore, the lattice algorithm used for MVAR coefficient estimation, while well suited for off-line analysis where all data is available for fitting, can be replaced by alternative estimation algorithms which take advantage of the convexity of MVAR model-fitting to compute robust models quickly and with relatively few data-points. In addition, the development of online recursive ICA (Hsu et al., 2014) allows for robust online artifact rejection, source identification, and lends to the overall feasibility of this approach in real-time BCI applications. There are other elements of the pipeline, such as the data-driven identification of ROI's for connectivity analysis, which must be adapted to account for the lack of all data present, or meet the computational demands in real-time analysis. Nevertheless, there is great potential use for this framework in both a clinical and research settings for real-time BCI control.

## Conclusion

The presented algorithmic and computational framework successfully identified a cortical network, across several subjects, associated with a cognitive task, using EEG signals recorded from individuals performing the task with the aid of a BCI. It also computed statistically significant causal dynamic connectivity profiles among the ROIs comprising the nodes of the identified cortical network. The modular nature of the presented framework allows for flexibility and scalability. The algorithm computing the cortical network can easily scale up to include a greater number of participants without compromising the reliability or accuracy. The ease of incorporating large amounts of data comes as a consequence of utilizing a distributed source localization method with a BEM-defined source space. The flexible-yet-fixed source space geometry allows for warping head models to align with a realistic 3D representation of each participant's cortex by way of the digitized electrode positions, while retaining the identity of anatomical ROIs and their associated gray matter grid points in the head model. Should the user deem it necessary, the modular nature of this framework allows for substitutions of preprocessing algorithms, source localization methods, and connectivity analysis tools as is seen fit. As long as a source-space based localization algorithm is utilized to maintain consistent anatomical regions across subjects, the user is free to create a customized pipeline that best suits their needs, and is not strictly limited to usage of the algorithmic pipeline with the proposed software and algorithms. The proposed analytical approach allows for extraction of information rich connectivity features which account for spectro-temporal connectivity dynamics during the performance of a cognitive task. This feature extraction is made possible by coupling the segmentation-MVAR modeling of cortical signals with the SdDTF—a connectivity metric that is evaluated independently for different frequencies, leading to the asymmetric spectro-temporal connectivity dynamics displayed in Figures 7–9. The presented framework also includes a non-parametric statistical test to assess the statistical significance of the extracted features—the spectro-temporal connectivity dynamics. The physiological plausibility of the cortical network and the causal dynamic features characterizing interaction among the nodes of the network computed by employing this framework strengthens the framework's applicability in reliably capturing complex brain functionality, which is required by applications, such as diagnostics and BCI.

## Ethics statement

This study was carried out in accordance with the recommendations of the Human Subjects Institutional Review Board of the University of California, San Diego with written informed consent from all subjects. All subjects gave written informed consent in accordance with the Declaration of Helsinki. The protocol was approved by the Human Subjects Institutional Review Board of the University of California, San Diego.

## Author contributions

HC introduced improvements to the pipeline, conducted re-processing and analysis of data, and wrote the manuscript. TM assisted with data processing on the first iteration of the pipeline. HP and GC contributed in an advisory manner with strategy and direction of the paper and the underlying computational methods. HP also directed the original project in which the data was collected, and GC assisting with editing the manuscript. JI (senior author) conducted processing and analysis on the first iteration of the pipeline, contributed in an advisory manner more closely than GC and HP, and assisted with editing the manuscript.

## Funding

This work was supported in part by the National Science Foundation (NSF) under Grant ENG-1137279 (EFRI M3C), Grant SMA-1041755 and Grant BCS-1460885, and in part by the Office of Naval Research (ONR) Multidisciplinary University Initiative (MURI) under Award No. N00014-10-1-0072.

### Conflict of interest statement

The authors declare that the research was conducted in the absence of any commercial or financial relationships that could be construed as a potential conflict of interest.
